# Resonant metasurface-enabled quantum light sources for single-photon emission and entangled photon-pair generation

**DOI:** 10.1515/nanoph-2025-0196

**Published:** 2025-09-05

**Authors:** Feng Pan, Priyanuj Bordoloi, Chih-Yi Chen, Jennifer A. Dionne

**Affiliations:** Department of Materials Science and Engineering, 6429 Stanford University, Stanford, CA, 94305, USA

**Keywords:** resonant metasurfaces, quantum light, heterogeneous integration, scalable quantum networks

## Abstract

Light encodes information in multiple degrees of freedom (e.g., frequency, amplitude, and phase), enabling high-speed, high-bandwidth communication through fiber optics. Unlike classical light, quantum light (single or entangled photons) can transmit quantum states over long distances without loss of coherence, thereby coherently interconnecting quantum nodes for distributed quantum entanglement. Quantum light sources are critical for developing scalable quantum networks aimed at distributed quantum computing, quantum teleportation, and secure quantum communications. However, existing quantum light sources suffer from limited integrability, insufficient spectral and spatial tunability, and inefficiencies in achieving mass-produced, deterministic, on-demand quantum light generation. These limitations significantly hinder progress toward direct, on-chip integration with quantum processing units and detectors – an essential step toward scalable quantum networks. Resonant metasurfaces that leverage photonic modes – such as Mie resonances, guided-mode resonances, or symmetry-protected bound states in the continuum – offer strong spatial and temporal confinement of electromagnetic fields, characterized by high quality factors and small mode volumes. These metasurfaces greatly enhance linear and nonlinear light-matter interactions, making them ideal for efficient on-chip quantum light generation and manipulation. Here, we describe recent advances in nanoscale quantum light sources and quantum photonic state manipulation enabled by resonant metasurfaces. We also provide an outlook on next-generation miniaturized quantum light sources achievable through materials innovations in quantum emitters, the co-design of resonant metasurfaces, and ultimately, the heterogeneous integration of emerging layered van der Waals materials with resonant metasurfaces.

## 1 Introduction

Quantum light sources (i.e. single or entangled photons) constitute critical building blocks for quantum communication [1], quantum computing [2], quantum metrology [3], and quantum sensing [4]. Unlike classical light, quantum light enables the transmission of quantum states over long distances without loss of coherence – critical for entanglement distribution between remote quantum nodes. Classical sources produce photons in groups or bunches with Poissonian statistics, making precise single-photon manipulation impossible and limiting their applications in quantum information science. In contrast, quantum light sources generate photons individually or in entangled pairs, which can carry and preserve quantum information with high fidelity and minimal disturbance. Single photons from these sources are indistinguishable and possess long coherence times, making them essential for encoding and transmitting quantum bits securely and reliably in quantum communication protocols. Complementarily, entangled photon pairs harness correlations beyond classical limits to enable advanced quantum information tasks such as quantum teleportation, quantum cryptography, and quantum computing.

Conventionally, both single photons and entangled photon pairs have been obtained using nonlinear crystals (e.g., beta-barium borate, potassium titanyl phosphate, periodically poled lithium niobate) with a typical thickness from millimeters to centimeters. High-purity, indistinguishable heralded single photons were detected by spectrally or spatially filtering twin photons [5], [6] emitted simultaneously from a nonlinear crystal. However, integrating these heralded single-photon sources into scalable quantum networks remains nontrivial due to their bulky size. In parallel, the spectral and angular tunability of entangled photon pairs is significantly limited by the stringent phase-matching condition and the resulting scarcity of suitable nonlinear materials [7]. The rise of III-V semiconductor quantum dots [8], [9] offers inherent integrability and scalability for generation of single or entangled photons; however, this comes at the expense of purity and indistinguishability in single-photon generation due to inhomogeneous broadening. Moreover, while the biexciton–exciton cascade process [10] enables deterministic entangled photon-pair emission, it restricts spectral tunability.

Ideally, quantum light sources could be integrated with on-chip circuits and detectors [11] in a compact and scalable footprint, to enable practical and widespread deployment of quantum information processing systems. The development of such miniaturized single-photon emission sources would facilitate the efficient generation of single photons with high purity and indistinguishability. Additionally, the relaxed phase matching condition in a miniaturized light source would enable the creation of multiphoton entanglement and biphotons in a continuous-variable basis across a wide range of frequencies and angles [7].

Metasurfaces are periodically arranged nanostructures with subwavelength thickness that control that phase, amplitude, frequency, and polarization of light, in both the near- and far-field [12]. When combined with other modes like Mie modes, guided-mode-resonances (GMR), or symmetry-protected bound states in the continuum (BIC) modes [13], such metasurfaces exhibit strong temporal and spatial confinement of electromagnetic fields, characterized by large quality factor (Q-factor) and small mode volume, respectively [14]. The resulting ‘resonant metasurfaces’ have a significantly increased electromagnetic local density of states that can enhance linear and nonlinear light–matter interactions. Such nanophotonic engineering opens the doors for the efficient generation of on-chip quantum light [15], [16], [17], [18], [19], [20]. For example, high-Q optical resonances strongly enhance spontaneous emission rates in quantum emitters and therefore facilitate single-photon extraction. Furthermore, wavefront shaping enabled by periodically arranged nanostructures results in far-field focusing of single-photon emission and steering of single photons at arbitrary angles. Similarly, resonant enhancement of nonlinear light–matter interactions favors entangled photon emission at designer-defined wavelengths for on-demand complex quantum states. Combined with polarization engineering, high-dimensional quantum entanglement would be possible in a compact platform.

**Figure 1:**
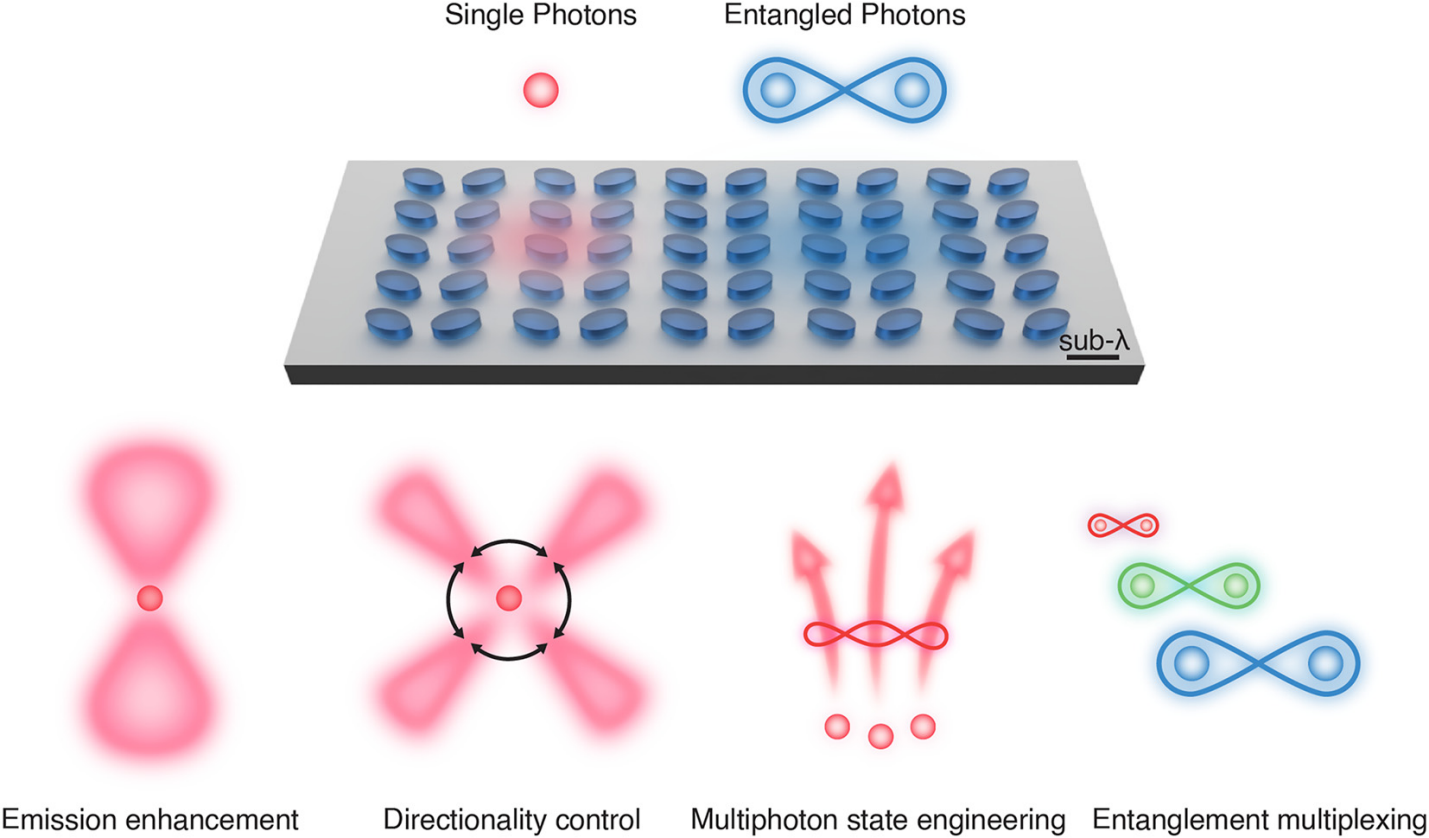
Resonant metasurface-enabled quantum light sources for single-photon emission and entangled photon-pair generation with engineered functionalities spanning emission enhancement, directionality control, multiphoton state engineering, and entanglement multiplexing. Note that the dimensions of the metasurface structure (e.g., unit cell, thickness, etc.) are on the subwavelength scale, as indicated by the scale bar, and the materials can be either dielectric (e.g. TiO_2_, SiN, GaAs, LiNbO_3_, etc.) or metallic (e.g. Au, Ag, etc.).

This *Perspective* summarizes recent advances in employing resonant metasurfaces to enhance and tailor single-photon and entangled photon-pair emission (Figure 1). Among recent advances, we discuss how resonant metasurfaces significantly enhance single-photon emission rates through deterministic coupling, enable cavity electrodynamics in the single-photon limit, provide control over emission directionality and polarization, and serve as powerful platforms for multiphoton state engineering and entanglement multiplexing. We also provide an outlook on next-generation miniaturized quantum light sources for scalable quantum networks. The field is especially poised to tackle integrability and scalability challenges and provide the broad spectral and angular tunability absent in conventional quantum light sources towards scalable quantum networks. We limit the scope of our review to state-of-the-art nanoscale quantum light sources and quantum photonic state manipulation enabled by resonant metasurfaces. For a broader understanding of metasurfaces for quantum photonics, interested readers are encouraged to refer to more comprehensive review articles [15], [16], [17], [18].

## 2 Resonant metasurfaces for enhanced and tailored single-photon emission

Single-photon emitters (SPEs) are evaluated based on the purity, indistinguishability, and efficiency of photon emission. An ideal single-photon source emits photons purely one at a time, produces indistinguishable photons that all carry the same quantum state and information, and operates at high rates and efficiencies [21]. Beyond the creation of single photons via heralded single photon sources, individual quantum emitters hosted in solid-state materials – including III-V semiconductor quantum dots (QDs) [22], monolayer transition metal dichalcogenides (TMDCs) [23], and defect-based color centers in diamond [24], silicon carbide [25], and hexagonal boron nitride (hBN) [26], – can generate on-demand streams of single photons (Figure 1). Despite significant progress in materials development and fabrication over the years, most SPEs are still plagued by low efficiencies inherent to the relatively weak spontaneous emission process [21], [27], [28].

In recent years, resonant metasurfaces have been shown to enhance quantum light–matter interactions and tailor emitted single photons via momentum engineering [15], [27], [28] (Figure 1). Table 1 summarizes the key figures of merit – namely, single-photon purity, emitter lifetime, and emission rate – for state-of-the-art demonstrations of single-photon emission enabled by resonant metasurfaces, spanning deterministic coupling, cavity quantum electrodynamics, and control of emission directionality and polarization. These structures strongly enhance optical transition rates (or decrease radiative lifetime) in quantum emitters, resulting in significantly improved quantum yields of up to 65 % [29] and SPE-metasurface strong-coupling at room temperature [30]. Demonstrations include heterogeneously integrated systems, where SPEs are coupled with metallic or dielectric resonant metasurfaces made of a different material [29], [30], as well as monolithic platforms, where SPEs are created directly within the resonant metasurfaces [31]. On-demand spin-state manipulation and multichannel emission of single photons have been achieved using anisotropic metasurfaces integrated with SPEs [32]. These metasurfaces enable complex control of quantum light emission and enhanced information capacity for high-dimensional quantum information processing. These advances may open new avenues for realizing ideal integrated SPE systems (see Table 1), enabling nearly perfect single-photon purity and bright emission with lifetimes in the target range of ∼0.1–1 ns and emission rates exceeding 100 MHz – critical metrics for practical quantum applications ranging from quantum computing and quantum sensing to quantum networking.

**Table 1:** Summary of the key figures of merit for state-of-the-art demonstrations of single-photon emission enabled by resonant metasurfaces. Note that the values in parentheses are intrinsic properties of single-photon emitters. The quantity of g^(2)^(0) represents the second-order photon correlation function evaluated at zero time delay, commonly used to characterize single-photon purity. QED: quantum electrodynamics.

Advancement or functionality	Ref.	Purity g^(2)^(0)	Lifetime (ns)	Emission rate (MHz)
**State of the art**				
Deterministic coupling	[29]	0.16 (0.21)	0.299 (13.7)	42
	[36]	0.04 (0.02)	0.42 (0.63)	0.89 (0.37)
Cavity QED	[30]	0.28	2.07	–
Polarization and/or directionality control	[32]	0.23	15 (40)	–
	[44]	0.129 (0.141)	21 (16)	–
	[45]	0.08 (0.16)	16 (19)	1.12 (1)
	[46]	0.2	1.35	–
**Ideal integrated system for practical quantum applications**				
Deterministic coupling, directionality and polarization control	–	0	−0.1–10 ns	>100

### 2.1 Toward brighter single-photon emission

When periodic nanostructures like nanopillars, nanocubes, or nanocones are integrated with monolayer TMDCs, the local lattice is deformed, inducing strain, resulting in the formation of trap excitonic states. This has enabled the deterministic creation of large-array SPEs [33], [34]. In combination with deterministic SPE creation via strain engineering, periodically arrayed plasmonic nanocavities placed atop monolayer TMDCs [29] significantly enhance radiative rates, leading to improved quantum yield (e.g., from 1 % to 65 %). The Purcell enhancement of transition rates is primarily attributed to the extremely small mode volume provided by plasmonic gap modes, although the Q-factors remain below 10. In contrast to strain engineering, SPEs in multilayer hBN are typically activated via high temperature annealing [35]. Tran et al. coupled lattice resonance modes in plasmonic nanocavity arrays to SPEs in hBN flakes, resulting in enhanced emission rates and reduced fluorescence lifetimes [36].

Resonant metasurfaces made of dielectric materials have minimal or negligible absorption loss compared to their metallic counterparts, and can achieve higher Q-factors through Mie modes [37] or quasi-BIC modes [38]. By leveraging high-Q quasi-BIC modes in TiO_2_ metasurfaces, Do et al. demonstrated strong coupling between SPEs in multilayer hBN and nonlocal optical modes at room temperature [30]. Sortino et al. reported a monolithic, scalable resonant metasurface composed of layered hBN, where spin-defect emitter ensembles, created via post-fabrication defect implantation, were coupled to high-Q quasi-BIC modes with Q exceeding 10^2^ [31]. This monolithic hBN platform may enable the direct creation of SPEs while utilizing quasi-BIC modes to achieve bright single-photon emission. Another monolithic quantum light source is color centers in silicon nitride (SiN) thin films grown on SiO_2_, generated via rapid thermal annealing [39]. This new type of SPE, offering bright, stable, high-purity quantum light at room temperature, has generated significant interest for the monolithic integration of SPEs with well-established SiN photonic platforms, and advances integrated quantum nanophotonics [40]. Despite great promise in this monolithic platform, achieving strong light–matter interaction between SPEs and optical modes requires site-controlled placement of SPEs within nanophotonic structures with sub-diffraction accuracy.

### 2.2 Tailoring single-photon emission

Designer-shaped nanostructures and their arrangement modify the amplitude, phase, and polarization of emitted photons. Integrating SPEs with these metasurfaces enhances single-photon collection efficiency, enables directional emission at arbitrary angles, and allows for the encoding of single photons with desired momentums (e.g., spin angular momentum (SAM), orbital angular momentum (OAM), linear momentum (LM)), as shown in Table 1.

For example, circular Bragg gratings shape the emission pattern in the far field while also enhancing the emission efficiency of single quantum emitters via an optical resonance, enabling more efficient collection of single photons [41], [42]. Furthermore, highly directional emission of single photons encoded with SAM has been demonstrated on a nanodiamond-embedded phase gradient metasurface. The varying width along the azimuthal direction imprints the specific SAM onto emitted single photons [43]. The mechanism involves a pump laser illuminating an embedded SPE, which is non-radiatively coupled to the surface plasmon–polaritons (SPPs) beneath the nano-ridge metasurface. The geometrically engineered phase through the nano-ridges enforces the emission of single photons with the desired handedness. Similarly, Liu et al. demonstrated the generation of single-photon circularly polarized vortex beams with different topological charges by arranging orthogonal nanorod dimers, coupled to SPEs via SPP waves, in a spiral or concentric pattern [44]. This approach achieves simultaneous control of SAM and OAM in quantum light. Such work opens new avenues for on-chip, high-dimensional quantum light sources, advancing quantum information processing.

Efficient separation of linearly [45] or circularly polarized single-photon emission is crucial for realizing multi-channel emission and simultaneously controlling polarization characteristics. This effect has the potential to increase information capacity for quantum information processing. Combining directional emission of single photons with SAM enables the on-demand generation and separation of spin states of emitted single photons. Bao et al. employed a bifocal metalens composed of geometrically oriented nanoblocks to direct the emission of single photons encoded with opposite SAMs, enabling propagation along arbitrary directions with high collimation [46]. Jia et al. demonstrated on-demand control of LM and SAM through anisotropic metasurfaces, achieving multichannel single-photon emission for momentum and spatial multiplexing in quantum communications [15].

## 3 Resonant metasurfaces for entangled photon sources

Materials that efficiently generate entangled photons are crucial components of quantum networks [47]. Several approaches to generate photonic entangled states exist, including spontaneous parametric down-conversion (SPDC), biexciton cascade process in quantum dots, and spontaneous four-wave mixing (SFWM) [48]. Of these approaches, SPDC is most commonly employed for producing entangled photon pairs. It is the reversed process of sum frequency generation (SFG) in which a single pump photon is down-converted into two correlated photons – signal and idler – through the spontaneous parametric amplification of vacuum thermal noise photons in a nonlinear medium. In this process, both momentum and energy are conserved $\omega_{p}=\omega_{i}+\omega_{s,};\vec{k_{p}}=\vec{k_{i}}+\vec{k_{s}}$ , where p, s, i refer to the pump, signal, and idler photons, respectively [49]. The stringent phase-matching condition limits the number of nonlinear materials that can be experimentally exploited for SPDC.

Beta-barium-borate (BBO) is commonly utilized as one of SPDC sources [50]. Its bulky size, accompanied by its relatively low nonlinear susceptibility tensor (*χ* ^(2)^ ≈ 2 pm/V) and fabrication challenges, prevents it from being miniaturized and subsequently integrated with photonic circuits for quantum information science [11]. Aluminum gallium arsenide (AlGaAs), gallium phosphide (GaP), and lithium niobate (LiNbO_3_) due to their *χ* ^(2)^ 1-2 orders higher than BBO, are the frontrunners of several nonlinear non-centrosymmetric materials for thin-film SPDC sources (Figure 1). Moreover, the phase matching condition becomes more readily satisfied in thin films due to the shortened interaction length. Okoth et al. (2019) demonstrated the first microscale SPDC source made of LiNbO_3_ crystal [7]. Unfortunately, the limited interaction length results in a low photon-pair generation rate, even in sub-micron scale thin films such as LiNbO_3_ and GaP with rates 0.32 Hz/mW and 0.06 Hz/mW, respectively [51], [52].

### 3.1 Nanophotonic engineering of nonlinear thin films for improved SPDC

The aforementioned nonlinear materials’ high refractive indices in conjunction with the development of nanofabrication techniques enable the creation of quantum nonlinear metasurfaces (QNMs) with high-Q optical resonances at pump and/or entangled photon emission wavelengths. A quantum–classical correspondence relationship states that the SPDC generation rate is proportional to its reverse classical SFG efficiency [53]. Thus, increasing SFG efficiency through enhanced electromagnetic local density of states at two pump wavelengths and SFG wavelength ultimately results in enhanced SPDC rate in a high-Q resonant metasurface. Doing so compensates for the limited down-conversion rate due to the significantly shortened interaction length in a nanoscale nonlinear medium.

Mie modes [54] are determined by both the refractive index contrast between the nano-scatterers and their surrounding medium and nano-scatterer geometry. These modes allow one to place electric or magnetic Mie-scattering resonances at pump and signal/idler emission wavelengths for enhancing the SPDC rate. Marino et al. were the first to create Mie-scattering-resonances at pump and signal/idler emission wavelengths in a crystalline AlGaAs nanocylinder with an absolute SPDC pair-generation rate of 0.01 Hz [55]. Similarly, a LiNbO_3_ metasurface consisting of truncated nanopyramids exhibited a rate of 1–5 Hz, featuring an enhancement of almost 130x compared to an unpatterned LiNbO_3_ film [56]. Along with enhanced generation rates, the signal/idler spectral linewidth can be controlled by detuning the Mie-scattering resonances from signal/idler emission wavelength. However, these resonances, usually with Q-factors less than 100 [57] in most dielectric materials, limit further improvements of SPDC rate.

GMR optical modes can also boost the SPDC rate in QNMs. Zhang et al. demonstrated a GMR-type QNM consisting of SiO_2_ nanogratings on the LiNbO_3_ thin film with Q-factors exceeding 100. They achieved an enhanced rate of 450x compared to unpatterned LiNbO_3_ thin film [58]. Another avenue is to take advantage of symmetry-protected BIC modes, which have infinitely large quality factors; however, they do not couple with external light. Introducing a small perturbation in the QNM lattice makes these modes leaky (i.e. q-BIC) while still having very high Q-factors. Using the extent of perturbation, q-BICs have been used to achieve Q-factors exceeding 10^4^ experimentally [59] for nonlinear enhancement. Recent experimental and theoretical demonstrations include q-BIC modes created by engineering the symmetries in GaP [60], GaAs [61], [62], [63], and AlGaAs [64], [65] QNMs such that electric- and/or magnetic-dipole q-BIC resonances are created at signal and/or idler emission wavelengths, respectively. The enhancement of SPDC rate is ultimately determined by experimentally achievable Q-factors in a QNM.

**Figure 2:**
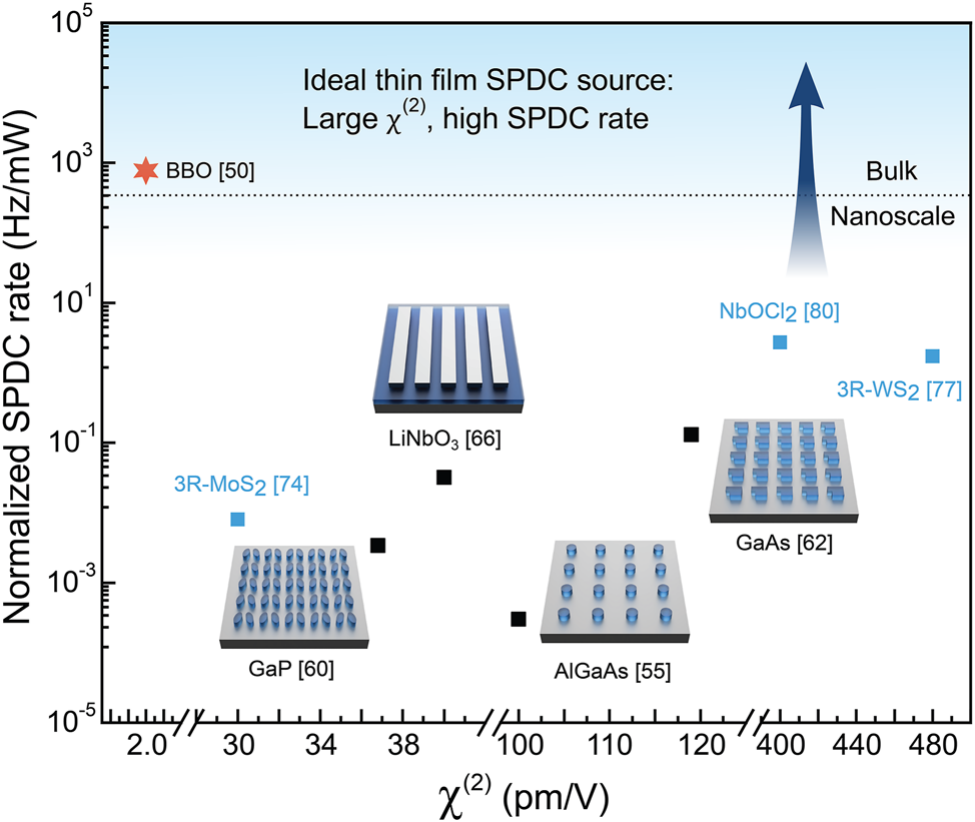
Experimental reported normalized SPDC rates for quantum nonlinear metasurfaces (black squares), layered van der Waals materials (blue squares), and a BBO bulk crystal (red star) versus materials’ nonlinear susceptibility (*Χ* ^(2)^). Note that the unnormalized SPDC rate (0.01 Hz) used here for Ref. [55] is lower than the reported value of 33 Hz, which accounts for losses from optical components and detectors.

Typically, SPDC rates reported in QNMs were compared to those determined in their unpatterned nonlinear thin-film counterparts. Despite significant advances driven by these metasurfaces, experimentally achieved rates (Hz) to date are still below the state-of-the-art BBO bulk crystal when normalized to pump power (mW). Figure 2 shows the highest SPDC performance experimentally demonstrated in QNMs versus their susceptibility tensor (*χ* ^(2)^), where the pair-generation rates are normalized by pump power. Clearly, SPDC rates demonstrated so far are still 2-3 orders of magnitude away from reaching those realized in the BBO platform, though most nonlinear materials exhibit significantly high *χ* ^(2)^ compared to BBO. Possible reasons could be limited experimentally achievable Q-factors below 1000 due to nanofabrication limitations and non-negligible absorption losses at visible wavelengths for pump photons in III-V semiconductor nonlinear materials.

### 3.2 QNMs for multifunctional engineering of entangled photon emission

While high-Q QNMs significantly enhance SPDC generation rate, entangled photon pairs generated by QNMs exhibit diverse emission directionality compared to phase-matched SPDC in bulk nonlinear platforms, including forward emission [61], backward emission [56], or bidirectional emission [60] with respect to the direction of pump photons. The reason is that the relaxation of phase matching in the nanoscale SPDC sources tolerates the mismatched longitudinal wave vector. Among these demonstrations, the bidirectional emission property enables heralded preparation of single photons through QNMs in which single photons are readily obtained without spatially or spectrally filtering twin photons. In parallel, angularly tunable photon-pair generation has been achieved in a GMR-type QNM consisting of SiO_2_ nanogratings on a LiNbO_3_ thin film. The underlying principle is the transverse phase matching of the GMR with strong angular dispersion [66]. Sweeping the pump wavelength results in a modified propagation constant along the guiding layer and thus tunes emission angles of signal and idler photons. Since the propagation constant is also dependent upon the refractive index, leveraging the strong Pockels effect in LiNbO_3_ enables electro-optic modulation of the directionality of entangled photon emission.

Polarization engineering is the path to creating entangled Bell states, along with spatial and frequency entanglement, leading to high-dimensional entangled photonic states. By tuning the orientation and structural symmetries of individual nano-scatters [12] in a QNM, we can exert facile control of polarization states in entangled photon pairs without modal coupling or additional optical components as required in their bulky counterparts [67], [68]. Ma et al. utilized GMR’s inherent polarization selectivity in a QNM made of orientation-multiplexed silica metagratings on LiNbO_3_ thin film to generate horizontally, vertically, and diagonally polarized entangled photonic states with fidelity of 89 %. This work lays the foundation for generating arbitrary polarization-entangled qutrit states [69]. Employing electric-dipole and/or magnetic-dipole q-BIC modes’ far-field polarization property, signal and idler photon emission can be imprinted with well-defined polarization states, resulting in nearly separable two-photon polarization states [63]. Engineered anisotropic susceptibility tensor via geometric symmetry breaking leads to the generation of a polarization-entangled Bell state with a fidelity of 0.91 [70]. A recent demonstration revealed that structural asymmetry in an InGaP QNM breaks the rotational symmetry of the nonlinear polarization and the polarization entanglement can be continuously tuned from near unentangled states to a Bell state by altering pump wavelength [71].

Additional functionality can be readily achieved in QNMs through quantum state engineering. The frequency-multiplexing via pumping a GaAs QNM at different wavelengths allows one to produce signal photons at the resonant wavelength and idler photons at multiple wavelengths due to the energy conservation, thus creating cluster states [61]. A high-dimensional and multiphoton quantum source has been demonstrated by integrating a metalens array with a thin cut BBO crystal. The metalens array enables two-, three- and four-dimensional two-photon path entanglement with fidelities over 95 % as well as four- and six-photon generation with high indistinguishability [72].

## 4 Summary and outlook

In this *Perspective*, we highlight recent advances in nanoscale quantum light sources and the manipulation of quantum photonic states enabled by resonant metasurfaces. The versatility of nanophotonic engineering through periodically arranged subwavelength nanostructures offers opportunities to strongly enhance single-photon emission, tailor quantum photonic states at will, boost SPDC efficiency, and access high-dimensional and multiphoton entanglement. These efforts position resonant metasurfaces as a platform for bright, stable, tunable, and scalable quantum light sources at room temperature, as well as for quantum state engineering of light emission across multiple degrees of freedom (i.e., polarization, momentum, directionality, and time-energy).

To date, single-photon generation and manipulation efforts have primarily focused on the visible and near-infrared spectral ranges. In parallel, significant attention has been given to the generation and manipulation of entangled photon pairs in the telecommunication band, partly because these photons can be transmitted through optical fibers with minimal loss, making them ideal for quantum networking. Meanwhile, entangled photons in the visible and X-ray range are highly desirable for quantum imaging applications. Expanding the spectrum of quantum light emission hinges on material innovation. With advancements in both SPE materials [73] (e.g. color centers in hBN or silicon carbide) and metasurface materials (e.g. diamond, silicon carbide, hafnium dioxide, etc.) beyond visible and near-infrared ranges, single-photon generation and manipulation may be extended across a broader spectral range. One wide-band-gap material, rhombohedral boron nitride [74], has recently been demonstrated to exhibit highly efficient entangled photon-pair generation in the visible band, underscoring the potential of advanced materials to extend capabilities of quantum photonics.

Two-dimensional (2D) layered van der Waals (vdW) materials have recently attracted significant attention as a new candidate for nanoscale quantum light sources [75]. *3R*-phase TMDCs exhibit a non-centrosymmetric crystal structure, i.e., lack inversion symmetry, resulting in a non-zero *χ* ^(2)^ value. Moreover, the thickness-independent broken inversion symmetry allows for scalable nonlinearity [76]. It has been recently shown that *3R*-phase WS_2_ and MoS_2_ thin films (e.g. 350 nm and 285 nm, respectively) display SPDC rates comparable to those demonstrated in QNMs (Figure 2). Additionally, maximally polarization-entangled Bell states have been obtained by leveraging materials’ crystal symmetry [77], [78]. In addition to *3R*-phase TMDCs, vdW NbOCl_2_ crystal with very high *χ* ^(2)^ has emerged as the thinnest SPDC source (<100 nm) reported so far [79] and vdW layer twist stacking enables the creation of polarization-entangled Bell states [80]. QNMs made of these vdW materials could combine their large susceptibility and high-Q resonances, potentially yielding a comparable SPDC rate to that in bulky BBO crystal. Despite the relatively larger susceptibility than those in III-V semiconductor materials, monolayer *3R*-phase TMDC as a SPDC source has not been demonstrated so far due to monolayer-limit interaction length. Integrating *3R*-phase monolayer TMDCs with high-Q resonant metasurfaces can compensate for limited interaction length and may be an exciting avenue for realizing the atomically thin SPDC source.

Broadly, the heterogeneous integration of 2D layered vdW materials with resonant metasurfaces could open new avenues for microwave-optical quantum transduction at single-photon level. Out-of-plane electric field trapping of excitons in monolayer TMDCs enable stable and tunable localized states [81], paving the way for super-radiant states through controlled exciton arrays toward on-demand photon generation. In parallel, resonant metasurfaces can be dynamically modulated in the few-gigahertz range – especially 4–8 GHz – which aligns with the energy-level transitions of superconducting qubits such as transmons. These heterogeneously integrated platforms serve as quantum transducers to interconnect superconducting qubits via single photons for distributed quantum computing. Entangled photons, as flying qubits, coherently link stationary qubits (e.g., atoms, ions, or superconducting circuits), facilitating distributed quantum computing and secure quantum communications. Mass-produced, deterministic, on-demand entangled photon generation enabled through heterogeneous integration may reach heralded-photon generation rates exceeding 100 MHz – a critical threshold for practical entanglement distribution across quantum networks.

In parallel, increasing the emission rate towards 100 MHz for practical quantum applications could be achieved through mode engineering in resonant metasurfaces to elevate Q-factors and enhance mode overlap between quantum emitters and the metasurface resonances. Recently a resonant metasurface with a record-high million-scale ultrahigh-Q GMR at near visible wavelengths [82], when integrated with monolayer WSe_2_, demonstrated laser-like, highly unidirectional, and narrow-linewidth exciton emission. This ultrahigh-Q GMR could be beneficial for controlling high-performance, coherent quantum light sources. The enhanced light–matter interaction enabled by such resonances not only improves emission rates but also increases directionality and coherence, which are critical for quantum communication and computing. Furthermore, the scalability and on-chip integrability of these metasurfaces offer a promising pathway toward compact, high-performance quantum photonic devices.

However, most reported metasurfaces for quantum light sources lack real-time tunability or reconfigurability due to their static nanostructure design. Reconfigurable optical metasurfaces have been demonstrated for classical light control across multiple degrees of freedom (e.g., directionality, polarization, wavefront, and emission rate). By leveraging dynamic reconfigurability in optically tunable materials – such as phase-change materials, liquid crystals, graphene, and nonlinear media [83] – applying external thermal, electrical, or optical fields could enable real-time manipulation of quantum states in quantum emitters integrated with these metasurfaces. These advances could provide opportunities for unprecedented control of quantum light emission. Dynamically tunable metasurfaces could serve as a critical platform for quantum modulators, switches, or gates, where deterministic and adaptive control of single-photon properties is required. Such capabilities are essential for advancing quantum information processing, enabling reconfigurable quantum networks, and interfacing diverse quantum systems on a single chip.
